# Role of Corneal Epithelium in Riboflavin/Ultraviolet-A Mediated Corneal Cross-linking Treatment in Rabbit Eyes

**DOI:** 10.1155/2013/624563

**Published:** 2013-06-27

**Authors:** Xiangchen Tao, Haiqun Yu, Yong Zhang, Zhiwei Li, Vishal Jhanji, Shouxiang Ni, Ya Wang, Guoying Mu

**Affiliations:** ^1^Department of Ophthalmology, Shandong Provincial Hospital Affiliated to Shandong University, No. 324, Jing 5 Road, Jinan, Shandong 250021, China; ^2^Department of Ophthalmology, Affiliated Hospital of Weifang Medical College, Weifang, Shandong 261000, China; ^3^Department of Visual Sciences and Ophthalmology, The Chinese University of Hong Kong, Shatin, Hong Kong; ^4^Centre for Eye Research Australia, University of Melbourne, Melbourne, VIC, Australia

## Abstract

*Purpose*. To evaluate the role of corneal epithelium in riboflavin/ultraviolet-A (UVA) mediated corneal collagen cross-linking treatment. *Methods*. Fifty New Zealand rabbits were divided into 5 groups: UVA treatment with or without corneal epithelium, UVA+riboflavin treatment with or without corneal epithelium, and control without any treatment. All rabbits were sacrificed after irradiation and subsequently 4 mm × 10 mm corneal strips were harvested for biomechanical evaluation. *Results*. UVA irradiation alone did not enhance the maximal stress and Young's modulus of corneal specimens with (3.15 ± 0.56 mpa, 1.00 ± 0.09 mpa) or without (3.53 ± 0.85 mpa, 0.94 ± 0.21 mpa) the corneal epithelium, compared to specimens in the control group (4.30 ± 0.68 mpa, 1.03 ± 0.24 mpa). However, UVA irradiation combined with riboflavin significantly increased the maximal stress and Young's modulus of corneal specimens with (5.27 ± 1.09 mpa, 1.23 ± 0.23 mpa, *P* < 0.05) or without (7.16 ± 1.88 mpa, 1.42 ± 0.16 mpa, *P* < 0.05) corneal epithelium when compared to the control group. The maximal stress and Young's modulus of cornea in UVA+riboflavin and “epithelium-off” group were 35.9% and 15.4% higher compared to the UVA+riboflavin and “epithelium-on” group, respectively (*P* < 0.05). *Conclusions*. Our study shows that UVA+riboflavin treatment significantly affects the biomechanical properties of the cornea with and without epithelial removal. However, corneas without epithelium seem to benefit more compared to corneas with the epithelium.

## 1. Introduction

Keratoconus is a noninflammatory, chronic progressive corneal disorder with an incidence of approximately 1 in 2,000 [1]. The clinical signs of keratoconus include stromal thinning followed by a cone-like protrusion of the cornea, which in turn induces progressive irregular astigmatism. In late stages of keratoconus, breaks in the Descemet's layer may lead to acute corneal hydrops which aggravates visual impairment. Common treatments included contact lenses and glasses, intracorneal rings, photorefractive keratectomy (PRK), and corneal transplantation [2, 3]. However, these treatment modalities do not retard or terminate the progression of keratoconus.

In 2003, Wollensak et al. reported riboflavin/ultraviolet-A- (UVA) induced collagen cross-linking (CXL) for stabilization of keratoconus [4]. In Wollensak's study, 70% of the eyes had a 2.01 diopters reduction in keratometry and 1.14 diopters reduction in refraction. An additional 60% of the eyes had a slight improvement in visual acuity. The transparency of the cornea and the lens, endothelial density, and intraocular pressure remained unchanged. The study demonstrated that cross-linking is a viable treatment modality that impedes the progression of keratoconus [4]. It was further demonstrated in an experimental model that riboflavin-UVA-induced collagen cross-linking led to an increase in mechanical rigidity in porcine corneas and an even greater increase in human corneas [5].

The conventional protocol for CXL is to remove the corneal epithelium before UVA irradiation. Previous studies [6–8] have demonstrated an enhancement of riboflavin penetration into the corneal stroma after removal of corneal epithelium. In the current study, we aim to determine the role of corneal epithelium in CXL by comparative evaluation of biomechanical properties of cornea after riboflavin/ ultraviolet-A-induced CXL treatment with or without corneal epithelium.

## 2. Methods

### 2.1. Animal Model

Fifty New Zealand rabbits (weight 2.0–2.5 kg, age 4-5 months), acquired from the Shandong Academy of Agricultural Sciences, were divided into 5 groups: UVA with (UVA epi-on) or without (UVA epi-off) corneal epithelium, UVA+riboflavin with (CXL epi-on) or without (CXL epi-off) corneal epithelium, and controls (without any treatment). The living environment of the experimental animals was maintained at 21°C with a 12-hour light and dark cycle. All rabbits were fed ad libitum. Animals used in this study were treated in accordance with the Shandong University Animal Experimentation Ethic Committee (AEEC) guidelines. The study protocol was approved by the AEEC. All treatments were performed on the left eyes only. 

### 2.2. Collagen Cross-Linking

The animals were anesthetized with intramuscular injection of 0.2-0.3 mL/kg xylazine (Hua Mu Animal Health Products, China) and intravenous injection with 0.5 mL diazepam (Jin Yao Amino Acid, China). Baseline thickness of the cornea was measured using a corneal pachymeter (DGH 550, DGH Technology Inc., USA). In the epi-off groups, central 8.5 mm corneal epithelium was scraped off with a scalpel. 0.1% riboflavin sodium phosphate was prepared by dissolving riboflavin sodium phosphate solution (Jiang'Xi pharmaceutical Co. Ltd., China) in distilled water. Distilled water was used instead of riboflavin in the control group. Riboflavin solution or distilled water (control group) was topically administered onto the cornea for a period of 30 minutes at an interval of 2 minutes. Subsequently, the cornea was illuminated with an ultraviolet light for 30 minutes using a UVA lamp (UVX 1000 system, IROC Innocross AG Co. Ltd., Switzerland) (wavelength 365 nm, irradiance 3.0 mW/cm^2^, total dose 5.4 J/cm^2^). Riboflavin administration was continued every 2 minutes during UV illumination. The same surgical procedure was undertaken in the epithelium-on group without removal of the corneal epithelium.

### 2.3. Evaluation of Corneal Biomechanical Properties

**Figure 1:**
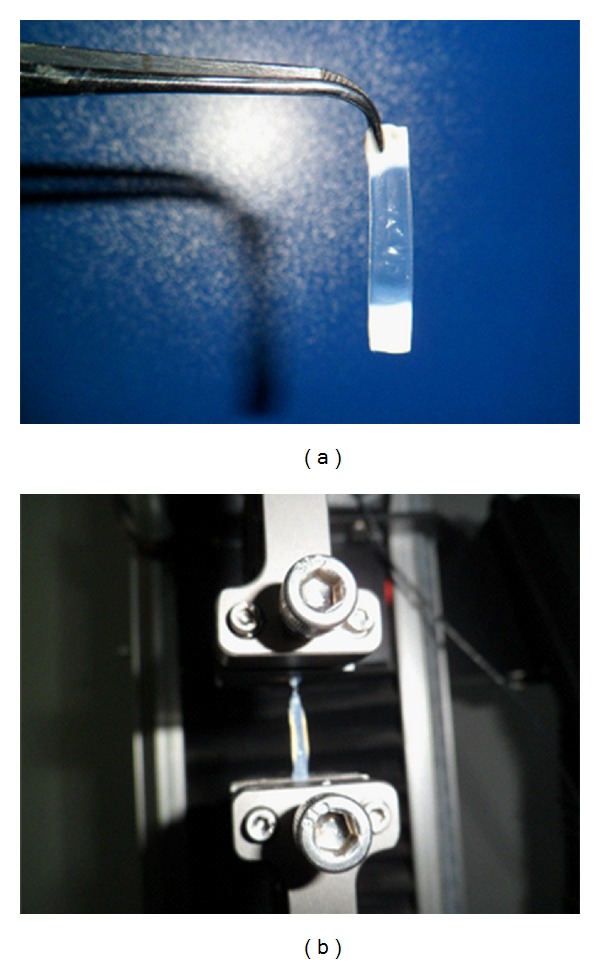
Evaluation of biomechanical properties of corneal strips was carried out on Instron 5544 system.

The rabbits were sacrificed by air embolism immediately after cross-linking procedure. For the preparation of the corneal strips, corneoscleral ring of the enucleated eyes was cut off in a circular manner and a 4.0 mm × 10.0 mm vertical corneal strip was removed from the 12 o'clock position with a scalpel. The corneal thickness was determined using a mechanical micrometer caliper. The corneal strips were clamped horizontally between the jaws of a commercially available microcomputer-controlled biomaterial-testing device with a distance of 8.0 mm (*L*_0_) between the jaws (Instron 5544 system, Instron Co. Ltd., USA) (Figure 1). The strain displacement was increased linearly at a rate of 2.0 millimeter/minute and was measured up to the point of tissue rupture. A curve for relationship between the load (*F*) and displacement (Δ*L*) was obtained. As suggested previously [9], the data in the range of 0–0. 04 N were selected to calculate the stress (*σ*) using the equation *σ* = *F*/4*t*, where *t* is the corneal thickness, and the related strain was obtained by *ε* = Δ*L*/*L*_0_. Young's modulus (*E*) was calculated by using the equation *E* = *dσ*/*dɛ* = *A* · *B*exp⁡(*B* × *ɛ*). The ultimate stress was measured at the tearing point. The ultimate strain was measured as a percentage of the starting length of the strip, represented by the amount of elongation at the point of tissue rupture.

Hematoxylin and eosin (H & E) staining was used to verify the success of epithelial removal and the presence of an intact Descemet's membrane.

### 2.4. Statistical Analysis

One way ANOVA was performed with MedCalc (Version 9.6.2.0) to compare the difference of maximal stress and Young's modulus between different groups.

## 3. Results

**Figure 2:**
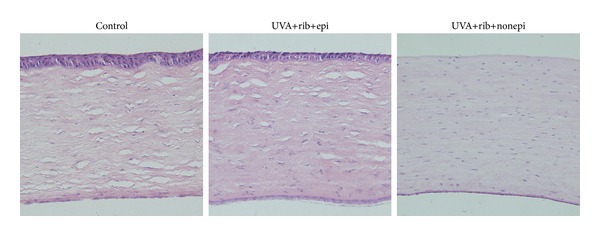
Histopathological analysis showed a successful removal of corneal epithelial cells in specified groups, with the integrity of Descemet's membrane (Hematoxylin eosin staining).

The average corneal thickness before epithelial debridement was 361 ± 15 *μ*m. There were no significant differences between the baseline central corneal thicknesses before UVA treatment in any of the groups. Histopathological analysis showed a successful removal of corneal epithelium in specified groups and an intact Descemet's membrane in all samples (Figure 2). 

**Figure 3:**
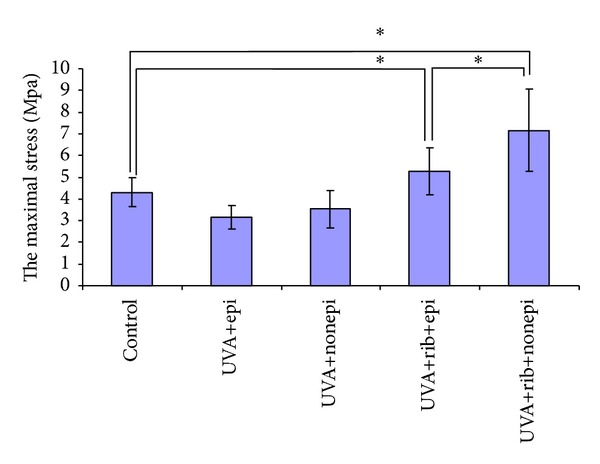
UVA irradiation alone did not enhance the maximal stress of cornea in epithelium-on (3.15 ± 0.56 mpa) or epithelium-off (3.53 ± 0.85 mpa) groups compared with those in control group (4.30 ± 0.68 mpa). The UVA irradiation combined with riboflavin significantly increased the maximal stress of cornea in epithelium-on (5.27 ± 1.09 mpa) or epithelium-off (7.16 ± 1.88 mpa) groups compared with those in control group (*P* < 0.05). The maximal stress of cornea in UVA+rib+nonepi group is 35.9% higher than that in UVA+rib+epi group (*P* < 0.05). Asterisk denotes significant difference with control group, unless otherwise specified.

**Figure 4:**
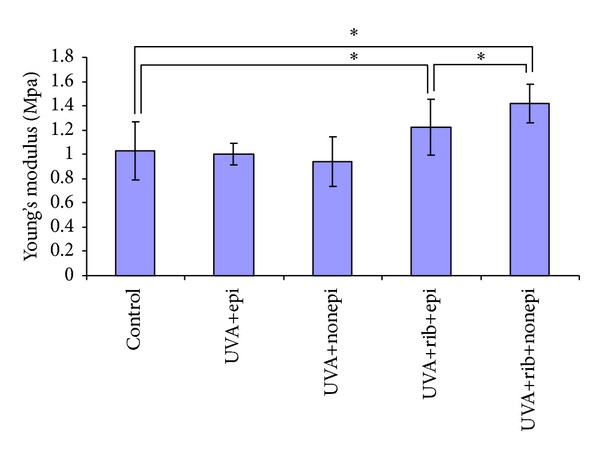
UVA irradiation alone did not enhance Young's modulus of cornea in epithelium-on (1.00 ± 0.09 mpa) or epithelium-off (0.94 ± 0.21 mpa) groups compared with those in control group (1.03 ± 0.24 mpa). The UVA irradiation combined with riboflavin significantly increased Young's modulus of cornea in epithelium-on (1.23 ± 0.23 mpa) or epithelium-off (1.42 ± 0.16 mpa) groups compared with those in control group (*P* < 0.05). Young's modulus of cornea in UVA+rib+nonepi group is 15.4% higher than that in UVA+rib+epi group (*P* < 0.05). Asterisk denotes significant difference with control group, unless otherwise specified.

UVA irradiation alone did not enhance the maximal stress and Young's Modulus of corneal specimens with (3.15 ± 0.56 mpa, 1.00 ± 0.09 mpa) or without (3.53 ± 0.85 mpa, 0.94 ± 0.21 mpa) corneal epithelium compared to the specimens in the control group (4.30 ± 0.68 mpa, 1.03 ± 0.24 mpa) (Figures 3 and 4). However, UVA irradiation combined with riboflavin significantly increased the maximal stress and Young's modulus of corneal specimens with (5.27 ± 1.09 mpa, 1.23 ± 0.23 mpa, *P* < 0.05) or without (7.16 ± 1.88 mpa, 1.42 ± 0.16 mpa, *P* < 0.05) the corneal epithelium compared to the control group (Figures 3 and 4). The maximal stress and Young's modulus of cornea in UVA+riboflavin+epithelium-off group were 35.9% and 15.4% higher compared to the UVA+riboflavin+epithelium-on group, respectively (*P* < 0.05). 

## 4. Discussion

As a soft biological tissue, cornea shares some properties of viscoelastic materials which can be evaluated by stress-strain curve, Young's modulus, and corneal hysteresis. Young's modulus is a major indicator of the elastic properties of cornea. It reflects the ability of a cornea to return to its original shape after being under stress. It has also been proposed to reflect the severity of corneal ectatic disorders such as keratoconus [10].

The last decade has seen a prominent increase in the popularity of CXL as a therapeutic measure to retard the progression of keratoconus. The stiffening of the upper 200–300 *μ*m of the corneal stroma is achieved through a photooxidative induction of collagen cross-links. The evidence of efficacy of CXL is supported by laboratory studies documenting the biomechanical and cellular changes induced by cross-linking [11, 12], as well as satisfying outcomes of CXL in the clinical practice [4, 13, 14]. Animal studies have elaborated that changes in the biomechanical properties of cornea after CXL were related to the irradiation time [15], light energy density, and riboflavin absorption [16], as well as animal species as evident by a greater increase of cornea rigidity in humans compared to porcine corneas [5]. Besides the optimized UVA and riboflavin solution parameters, the standard protocol for CXL [17] involves the removal of central 9 mm of corneal epithelium before riboflavin administration, and UVA irradiation. Recently, Kymionis et al. have demonstrated better visual and refractive outcomes after CXL combined with epithelial removal using t-PTK than after mechanical epithelial debridement [18]. 

However, considering the complications associated with the removal of corneal epithelium, most notably pain, risk of infection and postoperative corneal haze, an evaluation of the necessity of this step during CXL is desirable in clinical practice. Some surgeons have modified the standard protocol and performed the treatment without removing the epithelium [19–22]. In a study by Wollensak and Iomdina [23], there was a statistically significant increase in Young's modulus in corneas treated with standard CXL as compared to the epithelium-on corneas cross-linked with the addition of benzalkonium chloride-containing proxymetacaine eye drops. The authors proposed that corneal cross-linking without epithelial debridement reduced the biomechanical effect by approximately one-fifth compared to standard cross-linking probably due to restricted and inhomogeneous stromal distribution of riboflavin. Baiocchi et al. [24] showed that stromal concentrations of riboflavin increased with exposure time only if the corneal epithelium was removed. In our study we found a statistically significant increase in Young's modulus as well as the maximal stress in both epithelium- “on” and “-off” corneas. Further, we found that the maximal stress and Young's modulus of the corneal specimens in the epithelium-off group were 35.9% and 15.4% higher, respectively, than those in the epithelium-on group after riboflavin and UVA combination treatment. The enhanced effect of CXL in corneas without the epithelium is expected due to the absence of an intact epithelium in these eyes which would otherwise impair the absorption of riboflavin [7]. 

Kissner et al. [25] found that treatment with benzalkonium chloride 0.02% induces sufficient epithelial permeability for the passage of riboflavin as compared to the standard protocol. Raiskup et al. [26] demonstrated that addition of 0.01% benzalkonium chloride and 0.44% sodium chloride to riboflavin solution promotes its permeability through the epithelium, resulting in a sufficient concentration of riboflavin in the corneal stroma. In addition to the permeability issues of riboflavin through an intact corneal epithelium, the presence of epithelium represents a barrier for UVA penetration which is essential for a successful CXL procedure. It is possible that the UVA energy delivered through “epithelium-on” is different compared to the standard “epithelium-off” procedure [27]. 

In our study, the morphological analysis using H & E staining showed that the density of keratocytes and collagen fibers had no significant changes in CXL groups with or without corneal epithelium compared with that in control group (data not shown here). Mencucci et al. [11] reported that the diameter of corneal collagen fiber in CXL treated human corneal buttons increased by 22.6% compared with that in control group at 6 months postoperatively. In the present study, the corneas were harvested within 1 hour after CXL treatment, and therefore the pathological changes of cornea could not be identified with techniques used in our study. Considering the significant enhancement of corneal biomechanical properties in the very early stage after CXL, more specific and sensitive markers should be investigated to evaluate the pathological changes of cornea immediately after CXL treatment. Few clinical studies [28, 29] have attempted to evaluate the safety and efficacy of transepithelial CXL in cases with keratoconus. However, these studies are limited by small sample size, limited follow-up, and lack of comparative groups.

There are a few limitations of the present study. Corneas were retrieved early after the CXL procedure. Distilled water was used as a solvent for riboflavin in our study to match the control groups, as opposed to dextran which is being used in the commercial preparation of riboflavin. Application of hypotonic riboflavin solution without dextran during cross-linking procedure may alter the permeability of riboflavin and therefore lead to different results as compared to the standard riboflavin/dextran solution. Exposure of corneal epithelium to hyposmolar distilled water for 30 minutes may compromise the cell-cell tight junction integrity thereby potentially increasing permeability to the riboflavin solution [30]. This may explain a significantly improved biomechanical response compared with the control in our study even with an intact corneal epithelium (albeit less than that produced following epithelial removal). We did not evaluate the permeability of the riboflavin solution into the corneal stroma. Future studies can focus on evaluation of biomechanical properties of the corneas using other carriers which are being used for transepithelial CXL. It is noteworthy that the rabbit cornea lacks Bowman's membrane which is a possible barrier for the permeation of riboflavin during CXL procedure. Further studies should evaluate whether injury to the Bowman's membrane during epithelial removal will affect the outcome of CXL. Also, the rabbit corneas in their study were normal and not keratoconic.

Our study provides direct evidence of the effect of CXL procedure on corneal biomechanical properties. This effect was significantly enhanced after the removal of corneal epithelium during CXL.
